# Monitoring the durability of the long-lasting insecticidal nets MAGNet and Royal Sentry in three ecological zones of Mozambique

**DOI:** 10.1186/s12936-020-03282-w

**Published:** 2020-06-17

**Authors:** Ana Paula Abílio, Emmanuel Obi, Hannah Koenker, Stella Babalola, Abuchahama Saifodine, Rose Zulliger, Isabel Swamidoss, Gabriel Ponce de Leon, Eunice Alfai, Sean Blaufuss, Bolanle Olapeju, Hunter Harig, Albert Kilian

**Affiliations:** 1 National Institute of Health, Maputo, Mozambique; 2 PMI VectorWorks Project, Tropical Health LLP, Abuja, Nigeria; 3 PMI VectorWorks Project, JHU Center for Communication Programs, Baltimore, MD USA; 4 U.S. President’s Malaria Initiative, U.S. Agency for International Development, Maputo, Mozambique; 5 U.S. President’s Malaria Initiative, Centers for Disease Control and Prevention, Maputo, Mozambique; 6 U.S. President’s Malaria Initiative, Centers for Disease Control and Prevention, Atlanta, Georgia; 7 National Malaria Control Programme, Maputo, Mozambique; 8 PMI VectorWorks Project, Tropical Health LLP, Montagut, Spain

**Keywords:** LLIN durability, Monitoring, Mozambique

## Abstract

**Background:**

Malaria prevention with long-lasting insecticidal nets (LLINs) has seen a tremendous scale-up in sub-Saharan Africa in the last decade. To sustain this success, it is important to understand how long LLINs remain in the households and continue to protect net users, which is termed durability. This information is needed to decide the appropriate timing of LLIN distribution and also to identify product(s) that may be underperforming relative to expectations. Following guidance from the U.S. President’s Malaria Initiative, durability monitoring of polyethylene 150-denier LLIN (Royal Sentry^®^ and MAGNet^®^) distributed during a 2017 mass campaign in Mozambique was implemented in three ecologically different sites: Inhambane, Tete, and Nampula.

**Methods:**

This was a prospective cohort study in which representative samples of households from each district were recruited at baseline, 1 to 6 months after the mass campaign. All campaign LLINs in these households were labelled and followed up over a period of 36 months. The primary outcome was the “proportion of LLINs surviving in serviceable condition” based on attrition and integrity measures and the median survival in years. The outcome for insecticidal durability was determined by bio-assay from subsamples of campaign LLINs.

**Results:**

A total of 998 households (98% of target) and 1998 campaign LLIN (85% of target) were included in the study. Definite outcomes could be determined for 80% of the cohort LLIN in Inhambane, 45% in Tete, and 72% in Nampula. The highest all-cause attrition was seen in Nampula with 74% followed by Inhambane at 56% and Tete at 50%. Overall, only 2% of campaign LLINs were used for other purposes. Estimated survival in serviceable condition of campaign LLINs after 36 months was 57% in Inhambane, 43% in Tete, and 33% in Nampula, corresponding to median survival of 3.0, 2.8, and 2.4 years, respectively. Factors that were associated with better survival were exposure to social and behavioural change communication, a positive net care attitude, and folding up the net during the day. Larger household size negatively impacted survival. Insecticidal performance was optimal up to 24 months follow-up, but declined at 36 months when only 3% of samples showed optimal effectiveness in Inhambane, 11% in Tete and 29% in Nampula. However, 96% of LLIN still had minimal effectiveness at 36 months.

**Conclusions:**

Differences in median survival could be attributed at least in part to household environment and net care and repair behaviours. This means that in two of the three sites the assumption of a three-year cycle of campaign distributions holds, while in the Nampula site either continuous distribution channels could be expanded or more intense or targeted social and behaviour change activities to encourage net care and retention could be considered.

## Background

Malaria prevention with long-lasting insecticidal nets (LLINs) has seen a tremendous scale-up in sub-Saharan Africa in the last decade. Many countries have achieved high ownership coverage with LLIN following mass distribution campaigns combined with continuous distribution strategies, and are approaching the target of at least 80% LLIN access for the population at risk as recommended by the World Health Organization (WHO) [1]. A critical question now is how these successes can be sustained. In this context, it is important to understand how long distributed LLIN remain in the households and continue to protect net users, which is termed durability. This information is needed to decide the appropriate timing of LLIN distribution and also to identify product(s) that may be underperforming relative to expectations.

LLIN durability has two components, the insecticidal durability or effectiveness and the physical durability. Guidance of how to assess insecticidal effectiveness has been well established by the WHO since 2005 [2]. Physical durability in turn comprises of the loss of LLINs, attrition, and the physical condition of surviving LLINs, integrity. Recognition that the damage to LLINs could impact usefulness was published as early as 1982 [3], but attempts to capture the level of damage in early studies were poorly standardized, did not provide an overall metric of damage, and did not allow direct comparison of results [4–6]. A first suggestion of a more standardized approach was made in 2008 in the form of a Hole Index with three defined hole size categories [7]. In 2011, this was then extended to a proportionate Hole Index (pHI) that takes into account the relative size of hole categories [8] ]. Finally, further refinement of the pHI was done in the context of the WHO Vector Control Technical Expert Group, and a cut-off level of damage was agreed at which LLIN are no longer considered serviceable [9]. With this comprehensive guidance now available [1], the WHO recommends that all malaria control or elimination programs that distribute LLIN should also routinely monitor their durability ideally using a prospective study design. Other donors and implementation partners, such as the U.S. President’s Malaria Initiative (PMI), have taken up this recommendation and also encourage routine monitoring of LLIN durability in the countries they support.

Monitoring of LLIN durability in Mozambique is one of the priorities of the National Malaria Control Programme. Between 2008 and 2011, a first study on the durability of two types of LLIN was undertaken in Nampula Province comparing a 100-denier polyester, deltamethrin treated LLIN, PermaNet 2.0, to a 150-denier polyethylene, permethrin treated LLIN, Olyset over 3 years [10]. No difference was found in attrition between the LLIN types, but the results showed that early losses were mostly due to LLIN being given away to others to use in the first year. Losses due to wear and tear were low initially (5% of all losses) and then increased to 37% and 51% after 2 and 3 years. The study also found better performance of LLINs in households away from the coast (inland) compared to the coastal area. Preliminary analysis of residual insecticide efficacy showed that both Olyset and PermaNet LLIN retained their efficacy for 1 year of use in rural Mozambique. The efficacy gradually decayed and after 2 years only PermaNet insecticide residual efficacy was within the WHO recommendation of > 80% using the standard cone bio-assay to show optimal effectiveness [10].

The objectives of the present study were two-fold. First, to assess the physical and insecticidal durability of a 150-denier, polyethylene-based, alphacypermethrin treated LLIN in three locations in Mozambique with different environments over a three-year period and estimate median LLIN survival. Second, to compare the durability across the three locations and identify major determinants of field performance.

## Methods

### Study sites

**Fig. 1:**
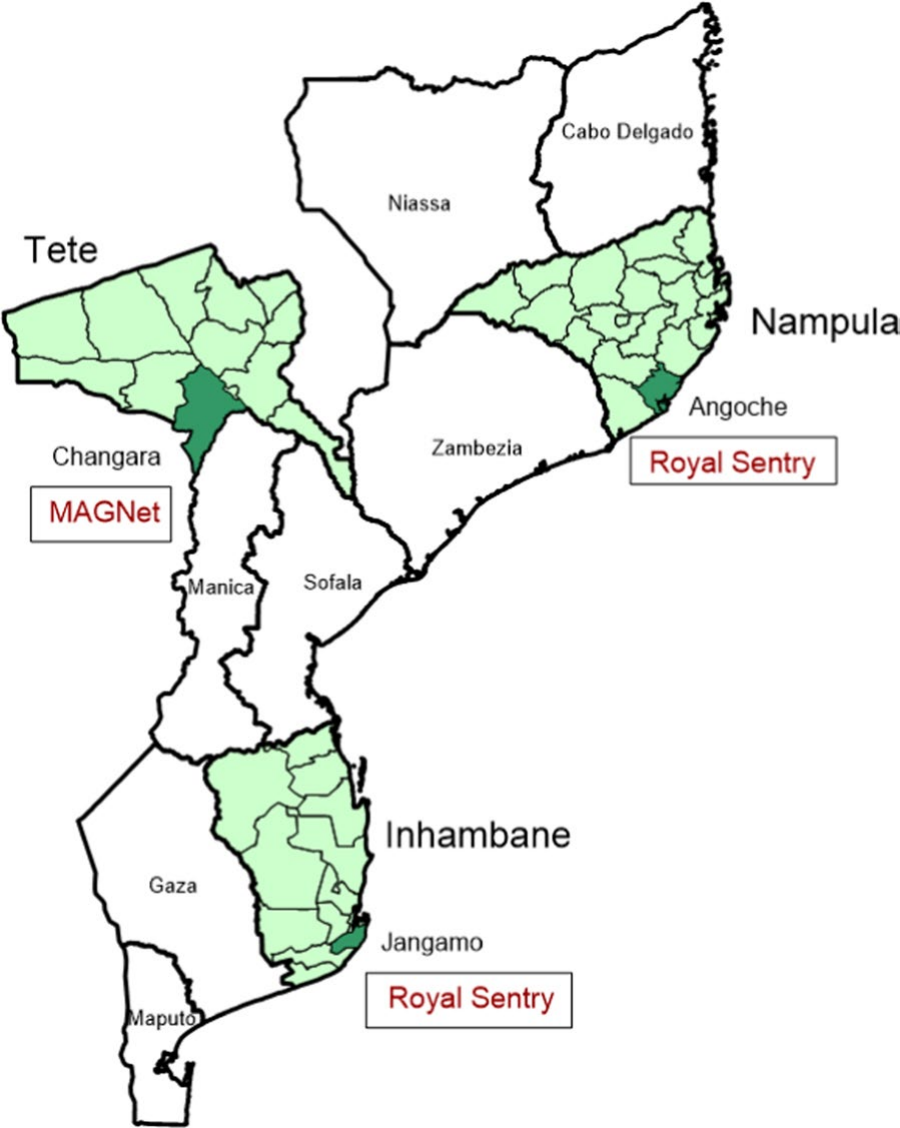
Location of study sites and tested LLIN brands within Mozambique

Three districts in three ecologically different provinces were purposively selected as the study sites based on timing of campaigns, malaria epidemiology, and environmental factors. Angoche district (Nampula province) is a coastal district located in the Northern region with high malaria transmission and has a population of 347,176 (based on the 2017 census). Changara district (Tete province) is located inland in the Central region with moderate to high malaria transmission and has a population of 123,056. Jangamo district (Inhambane province), located in the Southern region with moderate to high malaria transmission, is coastal and has a population of 105,306. Climate and ecology differ in the three sites. Temperatures are higher in the north and humidity higher in the coastal region. Throughout the country, the rainy season is from November to April, and average annual rainfall is around 1000 mm in Nampula, 800 mm in Inhambane and 650 mm in Tete. All three districts are mainly agricultural with subsistence farming and some fishing in the coastal communities in Angoche and Jangamo. The mass campaign for which this durability monitoring was carried out was undertaken in Tete in May 2015 using the MAGNet^®^ LLIN brand and in Inhambane and Nampula in October 2015, both distributing the Royal Sentry^®^ LLIN brand. An additional LLIN mass campaign was carried out in Nampula in September/October 2016. In addition, all sites were included in the national 2017 LLIN mass campaign which took place between the 24- and 36-month surveys in all three sites (Fig. 1).

### Study design

This was a prospective study of a representative cohort of LLINs distributed during the 2015 mass campaign and followed up for 3 years. The design was based on the guidance from the U.S. President’s Malaria Initiative for LLIN durability monitoring (www.durabilitymonitoring.org) and in this case the study compared the durability of two different LLIN brands with the same characteristics (150-denier polyethylene LLIN incorporating alphacypermethrin, in blue colour). Both products list a loading dose of 5.8 g/kg, obtained a full recommendation from the WHO Pesticide Evaluation Scheme (WHOPES) in 2011 [11], and were converted to WHO prequalification in late 2017 (Royal Sentry, #003-001) and early 2018 (MAGNet, #014-001). Within 6 months of the respective mass distribution campaigns, LLIN were sampled and followed up after 12, 24, and 36 months through household surveys. At each time, point measures of physical durability were assessed (attrition and integrity) using a household questionnaire and net damage assessment tools. For all data points after baseline, 30 campaign LLIN per site were sampled and retrieved for assessment of insecticidal effectiveness (bio-assay) and chemical analysis of the active ingredient.

### Sample size and sampling

Applying a design effect of 2.0 and 5% non-response rate of households, the required sample of LLIN after 3 years was 631 per site in order to detect a 12%-point difference between sites or estimate median survival of LLIN with a precision of ± 0.5 years (at alpha error 0.05 and beta error of 0.2). Taking into account the expected net attrition rates, a sample of 782 LLIN was estimated to be needed at baseline and based on the expected number of LLIN distributed per household (2.5), 340 households were needed to be sampled per site. These were sampled from 20 clusters (communities) with 17 households selected per cluster.

Clusters (communities) were sampled with probability proportionate to size using the campaign registration lists as sampling frame. Households within clusters were selected using simple random sampling from lists of eligible households prepared by the field teams on the day of the survey. For communities with more than 200 households a segmentation approach was used and only the selected segment was sampled. Up to five replacement households were sampled per cluster to substitute in case a sampled household had not received LLINs from the campaign or did not consent to participate. Within each household, all LLINs identified as from the campaign by brand, colour and report by the respondent were labelled with a unique ID number and bar-code for future follow-up, even when they were still in the package at the time of the baseline survey.

Campaign LLIN for bio-assay testing were sampled from the cohort (two LLIN per cluster) only at the final survey using simple random sampling. For the 12- and 24-months surveys, campaign LLINs were sampled from neighbouring households as follows: within each cluster two index households were randomly identified from the cohort and when the field teams reached these households, they went left to the next neighbour that had campaign LLIN and consented to give them up for the study. A brief questionnaire was filled for these LLIN regarding use and washing. For all LLIN collected for bio-assay new replacement LLIN were given.

### Field procedures

An implementation team of nine individuals was established per site, with one overall site coordinator and two field teams each consisting of one supervisor and three interviewers. Activities in the field were overseen by staff of the National Malaria Control Programme (NMCP) and the Mozambique National Institute of Health (NIH). Interviewers and supervisors were carefully selected so that they were culturally acceptable, had good knowledge of the local languages and experience in conducting household surveys.

A 5-day training was held at baseline and 3-day refresher trainings before each follow-up survey. Special emphasis was put on the process of a standardized assessment of net damage using a template to identify hole size categories and tallying hole counts using an application on the digital devices used for data entry. The questionnaire had three main modules: one for the household respondent, a second for the cohort campaign LLIN (including LLIN lost between campaign and baseline survey), and a third module for other LLIN owned by the household at each time point. In addition, a list of household members and assets was obtained at baseline and at the final survey. GPS coordinates were recorded at baseline and used to track household during follow up. If households moved within the clusters the new homes were identified, if they moved outside the cluster, they were considered lost to follow-up.

The baseline assessment took place in October–November 2015 in all three sites. The 12-month survey was done in Tete in June 2016, and in Nampula and Inhambane in August 2016. The 24-month follow-up took place in May 2017 in Tete and August 2017 in Inhambane and Nampula. The 36-month final follow-up was done in May 2018 in Tete, and July/August in Inhambane and Nampula.

### Laboratory analysis

Outcomes of insecticidal effectiveness were based on bio-assay results using the standard WHO cone test, carried out at the Mozambique NIH in Maputo. A pyrethroid-sensitive strain of *Anopheles arabiensis* was used with 10 mosquitoes per cone, five sites tested on each net (four sides and roof) and four replicates per location (20 cone tests with 200 mosquitoes per net in total). Recorded were 60-min knock-down (KD60) and 24-h mortality and then combined as optimal insecticidal effectiveness (KD60 ≥ 95% or functional mortality ≥ 80%), minimal effectiveness (KD60 ≥ 75% or functional mortality ≥ 50%), or failure (not reaching minimal effectiveness criteria) [9]. Chemical residue analysis was done at the Centers for Disease Control and Prevention in Atlanta, Georgia. Five pieces of netting were tested per net from the same locations as for bio-assays and the fabric weight per surface area recorded. The five samples were then cut into 10cm × 10cm squares and pooled to get a homogeneous sample per net. The active ingredient (AI) incorporated into the filaments was extracted by heating under reflux for 30 min with xylene in presence of citric acid, addition of dioctyl phthalate as internal standard, and determination by gas chromatography with flame ionization detection (GC-FID) following the Collaborative International Pesticide Analytical Council (CIPAC) method 454/LN/M/3.2 [12].

### Data management

For data collection, tablet PCs (Samsung Galaxy Tab 5) were used and installed with the Open Data Kit (ODK) software for the questionnaire and Open Street Map for Android (OSMAND) for household tracking. Data from each field team was collected daily and directly uploaded to a secure data base if internet was available or collected on a local storage device by the coordinator until it could be transferred. Data was converted from ODK to comma-delimited data files using the ODK briefcase tool for inspection of incoming data and daily feedback was provided to the teams. For each survey round updated lists were compiled from the household and cohort net master files and preloaded on the ODK system including all households and cohort LLIN for which no definite outcome was available to date. After completion of the surveys, datasets were transferred to Stata version 14.2 (Stata, Texas, USA) for further aggregation, consistency checks and preparation for analysis. Stata do-files (macros) developed by the PMI VectorWorks project were applied and adjusted as needed [13]. For the final analysis data sets from all four surveys were merged and a duration format data set prepared for survival analysis.

### Definition of outcomes

The primary outcome measure was the physical net survival and was defined as the proportion of cohort LLIN received from the LLIN campaign still in serviceable physical condition (definition provided below) [14]. Physical net survival incorporates both net attrition and net integrity, which were calculated as follows:

Net attrition rate due to wear and tear was defined as the proportion of originally received LLIN which were lost due to wear and tear (thrown away, destroyed or used for other purposes) at the time of assessment. LLIN received but given away for use by others or stolen were excluded from the denominator. Similarly, LLIN with unknown outcome were excluded.

Net integrity was measured first by the proportionate Hole Index (pHI) as recommended by WHO [15]. Holes in cohort LLIN were counted categorized into four different sizes: size 1: 0.5–2 cm, size 2: 2–10 cm, size 3: 10–25 cm and size 4: larger than 25 cm in diameter. The proportionate Hole Index (pHI) for each net was then calculated as the number of holes counted multiplied by the size category weights as suggested by the WHO [15]. Based on the pHI each net was then categorized as “good”, “damaged”, “serviceable” or “torn” as follows [15]:

Good: total hole surface area < 0.01 m^2^ or pHI < 64.

Damaged: total hole surface area 0.01–0.1 m^2^ or pHI 65–642.

Torn: total hole surface area > 0.1 m^2^ or pHI > 642.

Serviceable: total hole surface area ≤ 0.1 m^2^ or pHI ≤ 642 (good or damaged).

In order to be able to compare physical survival measured at different time points the outcome of median net survival was estimated defined as the time in years until 50% of the originally distributed LLIN were no longer serviceable. Two approaches were used to estimate median survival. At each time point, the proportion surviving in serviceable condition were plotted against the hypothetical survival curves with defined median survival [14] (Additional file 1) and the median survival was taken as the relative position of the data point on a horizontal line between the two adjacent median survival curves. After the final survey median net survival was calculated from the last two time points provided both were below 85% (when the hypothetical curves are linear), using the following formula:

$${\text{tm}} = {\text{t}}1 + \frac{{\left( {{\text{t}}2 - {\text{t}}1} \right) *\left( {{\text{p}}1 - 50} \right)}}{{\left( {{\text{p}}1 - {\text{p}}2} \right)}}.$$

where tm is the median survival time, t1 and t2 the first and second time points in years and p1 and p2 the proportion surviving to first and second time point respectively in percent. Confidence intervals for this estimate were calculated by projecting the 95% CI from the survival estimates in the same way as described above.

### Explanatory variable preparation

Overall household attitudes towards net care and repair were measured using a set of Likert score questions where a statement was read to the respondent (head of household or spouse) and the level of agreement recorded. These were analysed by recoding the four-level Likert scale score to have a value of -2 for “strongly disagree”, -1 for “disagree”, +1 for “agree” and +2 for “strongly agree.” These attitude scores for each respondent were then summed and divided by the number of statements to calculate an average household attitude score for which 0 represents a neutral result and positive values a positive result. For each site the proportion of households with a score above 1 (very positive attitude) were calculated at each survey. Further aggregation of results was done across all four surveys to determine whether a household was never found to have a very positive attitude score, at least once or twice or more. Results were aggregated across all four surveys i.e. “never” = responded with “never” in all surveys the household participated; “at times” = household reported the behaviour as “sometimes” in at least one survey round or had conflicting statements; “always” = responded with “always” in all surveys the household participated. Exposure and attitude were similarly aggregated, i.e. “once” = reported exposure or positive attitude score at one of the four survey rounds; “twice or more” = at two or more survey rounds. The same procedure was used for other household and net risk factors for durability.

A wealth index was calculated for the baseline data set using the basic household assets and a principal component analysis with the first component used as the index. Households were then grouped into tertiles. The full household data collection and wealth index was repeated at the final survey. However, at 12 and 24 months no specific household or member data were collected.

### Statistical analysis

For continuous variables, arithmetic means were used to describe the central tendency and the *t* test for comparison of groups for normally distributed data. Otherwise, median and Kruskal–Wallis test were used. Proportions were compared by contingency tables and the Chi squared test used to test for differences in proportions. For calculation of confidence intervals around estimates, the intra- and between-cluster correlation has been taken into account.

Survival analysis was done using an intention to treat approach, i.e. risk of failure was considered to start at the day of distribution irrespective of whether or when the net was hung and used. Failure was defined as a net being lost to wear and tear or “too torn” based on physical assessment. Nets that were given away or with unknown outcome were censored. The time of failure was directly calculated from the report of time of loss by the respondent or taken as the mid-point between the last two surveys if unknown. A secondary analysis used a per-protocol approach where the risk of damage was considered to begin only when a net was first hung. Determinants of survival were explored using Cox proportionate hazard models. Final model fit was tested using a link test and Schoenfeld residuals to check the proportionate hazard assumption.

## Results

### Sample

At baseline a total of 998 households (98% of target) were recruited and 1988 campaign LLIN labelled for follow up, 726 in Inhambane (93% of target), 601 in Tete (77%) and 661 (85%) in Nampula. The slightly lower recruitment rates in Tete and Nampula were due to a lower household size than expected. Table 1 summarizes the follow-up status of households and cohort LLIN. The most important reasons for loss of households in Nampula and Inhambane was that they had lost all their labelled LLIN meaning no further follow-up was needed. In contrast, most common reason for loss in Tete was households not present as families there spent extended time at far away farms or in mining areas. In addition, during the last survey two clusters could not be reached by the field teams due to flooding of the roads. The second most common reason was households moving away from the community. This was particularly high in Nampula with 18% of households. Only 17 households refused participation (2%), eight in Tete and nine in Nampula. Due to the high absentee rate in Tete, the proportion of cohort LLIN with a definite outcome there was only 45% compared to 72% in Nampula and 80% in Inhambane. At 36 months, data from two clusters in Tete were excluded from final analysis due to concerns related to data quality.

**Table 1:** Follow-up status of households and campaign cohort LLIN at final survey

Variable	Inhambane	Tete	Nampula
Households	N = 340	N = 333	N = 325
Still has any campaign LLIN	54.1 (48.3–59.9)	36.3 (27.4–46.4)	34.5 (24.7–45.8)
Lost all their campaign LLIN	28.8 (22.3–36.3)	16.5 (10.8–24.4)	39.1 (28.8–50.4)
Moved away	8.2 (6.3–10.7)	9.9 (6.6–14.7)	17.5 (14.4–21.3)
Refused	0.0	2.4 (1.2–4.7)	2.8 (1.2–6.2)
Nobody home at survey or not reached	8.8 (5.9–13.0)	34.8 (24.2–47.2)	6.2 (3.4–10.9)
Campaign cohort LLIN	N = 726	N = 601	N = 661
Known outcome	79.8 (74.9–83.9)	44.9 (34.1–56.3)	72.0 (64.4–78.5)
Unknown outcome	20.3 (16.2–25.1)	55.1 (43.7–65.9)	28.0 (21.5–35.6)
Household moved away or refused	7.2 (5.5–9.3)	11.8 (7.8–17.5)	19.5 (15.2–24.7)
Net used elsewhere	0	3.5 (1.2–9.4)	0.2 (0.0–1.1)
Fate of net unknown	13.1 (9.3–18.1)	39.6 (28.0–52.6)	8.3 (5.4–12.6)

### Socio-demographics

Comparing households that participated in both the baseline and 36-month surveys (N = 550) the data was explored for differences between provinces (Additional file 1). Household size was significantly larger in Inhambane (5.0) than in Tete (4.0) or Nampula (4.1). Inhambane also had slightly older and more educated heads of household, and more female-headed households.

Population age structure was similar. There was a clear difference between the three sites across all indicators showing that Jangamo district in Inhambane was economically significantly better off than the other two sites. Other indicators confirm the differences between sites. Access to safe water was 100% in Inhambane and 79% and 89% in Tete and Nampula respectively. Any form of latrine was available for 97% of households in Inhambane compared to 67-75% in the other two sites. Ownership of any phone was higher in Inhambane (71%) and Nampula (58%), but lower in Tete (29%) where in some parts of Changara District there is no coverage. Similarly, ownership of smartphones was 18% in Inhambane and only 4% in Tete and 1% in Nampula. A similar difference was seen for other “luxury” household assets such as television (40% vs. 7% and 15% respectively), refrigerator (17% *vs.* 5% and 5%, respectively) and fan (12% *vs*. 3% and 4%, respectively). There was no evidence of a significant change in the socio-economic status of households during the study period.

Quality of housing was more similar with mainly thatch or grass roofs, but the wealth difference can be seen in the floor materials. In Inhambane 78% of houses had floors made from improved materials compared to 15% in Tete and 18% in Nampula.

### Determinants of durability

Factors that have previously been shown to be associated with LLIN durability were explored. These can be divided into factors of the net use environment in the household, knowledge and attitudes towards net care and repair of the household respondent, net handling and washing, and type of sleeping place. Household-level factors depended on the information provided by the respondents and these were in Nampula in 66% the head of household, 29% the spouse and 5% other adult household members. In Tete the respective rates were 57%, 37% and 7% and in Inhambane 42%, 43% and 15%, respectively.

Perceived presence of rodents was generally very high and highest in Nampula where at least 90% of household respondents were aware of the presence of rodents at all time points, followed by Inhambane with a consistent reporting of rodent presence by three quarters of respondents. Only in Tete was there some variation with lower values at baseline (48%) and at 12 months (29%) and increased reported rodent presence in the last two surveys (62% and 71%).

Other household level factors were calculated across all surveys and are presented in Table 2.

**Table 2:** Net use environment at household level across all survey rounds

Variable	Inhambane	Tete	Nampula	P-value for site comparison
Households	N = 245	N = 132	N = 173	
	% (95% CI)	% (95% CI)	% (95% CI)	
Storing of food in sleeping rooms				P = 0.001
Never	41.3 (33.7–49.3)	41.2 (29.7–53.7)	10.1 (4.7–20.2)	
At times	54.7 (47.6–61.6)	54.9 (44.3–65.2)	55.4 (43.7–66.5)	
Always	4.1 (2.2–7.5)	3.9 (2.0–7.2)	34.5 (23.1–48.1)	
Cooking in sleeping room				P = 0.001
Never	76.1 (62.9–85.7)	45.6 (33.8–57.8)	18.2 (7.7–37.2)	
At times	22.1 (13.7–33.7)	43.6 (33.7–54.1)	58.7 (42.6–73.1)	
Always	1.8 (0.6–5.4)	10.8 (6.0–18.7)	23.1 (11.9–40.1)	
Exposure to net use or care messages				P = 0.001
Never	9.2 (4.4–18.4)	36.4 (25.9–48.3)	20.9 (13.2–31.4)	
Once	17.4 (11.8–24.9)	36.5 (28.3–45.6)	39.1 (32.2–46.5)	
Twice or more	73.4 (60.1–83.5)	27.1 (16.7–41.0)	40.0 (30.4–50.5)	
Very positive net care attitude (score > 1.0)				P = 0.012
Never	62.6 (42.1–79.3)	63.8 (53.5–73.0)	40.7 (30.3–52.1)	
Once	17.2 (11.5–34.2)	30.9 (23.6–39.2)	35.3 (29.8–41.1)	
Twice or more	20.2 (9.6–37.6)	5.3 (2.9–9.6)	24.0 (14.4–37.2)	

Results were aggregated across all four surveys i.e. “never” = responded with “never” in all surveys the household participated; “at times” = household reported the behaviour as “sometimes” in at least one survey round or had conflicting statements; “always” = responded with “always” in all surveys the household participated in. Exposure and attitude were similarly aggregated, i.e. “once” = reported exposure or positive attitude score at one of the four survey rounds; “twice or more” = at two or more survey rounds

Storing food in the sleeping room is thought to attract rodents and thereby increases the potential damage of LLIN by rodents. Across the four surveys, this practice was less common in Inhambane or Tete with 40% of households never reporting doing this in any of the surveys they were interviewed and 4% always reported it, while in Nampula only 10% of households never stored food in sleeping rooms and 35% reported always doing so in all surveys they were interviewed.

Cooking in the same room where LLIN are hanging is a potential source of heat damage (melting of polyethylene yarn), especially if the cooking fuel is firewood or charcoal as was the case for 99% of all enrolled households. This practice was again very uncommon in Inhambane, moderately common in Tete, but was reported much more frequently in Nampula.

Recall of messages heard or seen in the last 6 months about net use or care and repair was low in Tete and Nampula, with only 27–40% of households recalling hearing net care messages at two or more surveys, but was better in Inhambane at 73%. The household care and repair attitudes were generally low with less than a quarter of households having a very positive attitude at any time during the 3 years.

Net-level factors are presented in Table 3. At least two-thirds of cohort LLINs were observed hanging at some point during the following up, and 60–70% were reported used with no difference between sites. The proportion of cohort LLIN that were hanging loose over the sleeping place and were not folded up or tied during the day was consistently high in Inhambane with nearly 90% and 75% of them never tied up over the 3 years in Inhambane and Nampula, respectively, but in Tete over a third of LLINs were always tied up and another 20% sometimes tied up (p < 0.0001 for site comparison). The cohort LLINs were mostly used over reed mats in Tete (93%), and finished bed frames in Inhambane and Nampula (around 40%). Mattresses were rare in all sites.

**Table 3:** Net use environment and washing of cohort LLIN from campaign across all survey rounds

Variable	Inhambane	Tete	Nampula	P-value for site comparison
	% (95% CI)	% (95% CI)	% (95% CI)	
Cohort LLIN	N = 737	N = 619	N = 675	
Ever hung	65.7 (61.4–69.7)	75.0 (65.3–82.7)	70.2 (63.2–76.4)	0.18
Ever used	62.8 (58.5–67.0)	70.6 (63.0–77.2)	69.6 (62.7–75.8)	0.15
Cohort LLIN ever hung	N = 484	N = 464	N = 474	
Tied up or folded when hanging				<0.0001
Never	88.0 (83.3–91.6)	43.1 (35.0–51.6)	74.1 (61.9–83.4)	
At times	8.1 (5.3–12.0)	19.8 (14.7–26.3)	19.2 (12.3–28.7)	
Always	3.9 (2.2–7.0)	37.1 (30.8–43.8)	6.8 (3.0–14.8)	
Type of sleeping place**				<0.0001
Bed frame (finished)	40.2 (32.8–48.1)	0.9 (0.4–2.2)	42.8 (30.9–55.6)	
Bed frame (sticks)	26.1 (20.2–32.9)	1.6 (0.5–4.8)	23.7 (17.2–31.7)	
Foam mattress	4.1 (2.3–7.0)	4.3 (0.8–19.9)	2.0 (0.7–5.6)	
Reed mat	29.7 (23.8–36.4)	93.3 (81.2–97.8)	31.6 (20.6–45.1)	
Cohort LLIN ever used	N = 445	N = 430	N = 461	
Net was used only by:				< 0.0001
Children	17.5 (14.1–21.5)	18.1 (13.5–23.9)	15.4 (11.7–20.1)	
Children with adults	15.5 (11.4–20.8)	52.1 (43.4–60.7)	20.4 (16.3–25.2)	
Adults	67.0 (59.7–73.5)	29.8 (24.8–35.3)	64.2 (58.2–69.8)	
Ever washed	87.3 (84.1–89.9)	76.2 (58.9–87.7)	68.1 (58.1–76.6)	0.03
Cohort LLIN ever washed	N = 437	N = 373	N = 322	
Washes last 6 months	1.5 (1–2)	2.6 (2–4)	2 (1.5–3.5)	< 0.0001
Median (IQR)				
Use of detergent				< 0.0001
Never	77.6 (68.9–84.4)	36.5 (26.9–47.3)	74.5 (63.2–83.3)	
At times	15.6 (10.9–21.7)	24.9 (16.9–35.2)	16.2 (10.1–24.8)	
Always	6.9 (4.3–10.8)	38.6 (28.7–49.6)	9.3 (4.4,18.5)	
Drying net outside				0.31
Never	3.2 (1.3–7.5)	2.1 (1.0–4.7)	0.9 (0.3–2.6)	
At times	7.6 (3.0–17.7)	6.2 (3.3–11.1)	3.1 (1.2–7.6)	
Always	89.2 (76.8–95.4)	91.7 (86.6–95.0)	96.0 (91.9–98.0)	
Drying over bush or fence				< 0.0001
Never	62.5 (50.0,73.5)	33.5 (23.0–45.9)	58.4 (42.5–72.7)	
At times	19.5 (12.8–28.5)	24.7 (18.3–32.4)	18.0 (11.5–27.1)	
Always	18.1 (12.6–25.2)	41.8 (30.9–53.6)	23.6 (13.1–38.8)	

** most rudimentary type of sleeping place ever reported for net

Across the four survey rounds, the majority of LLINs were used only by adults for Inhambane and Nampula, but there was a higher proportion of LLINs shared by adults and children in Tete.

The proportion of cohort LLINs reported ever washed after 36 months was 87% in Inhambane, 76% in Tete and 68% in Nampula. The washing frequency showed some variations but was an average rate of about two washes every 6 months at all three sites. The proportion of households reporting washing with a detergent was overall low in Inhambane and Nampula and moderate in Tete with 38% always using detergent. Nearly all LLIN were reported to be dried outside and drying on fences or bushes was somewhat more common in Tete than the other sites.

**Fig. 2:**
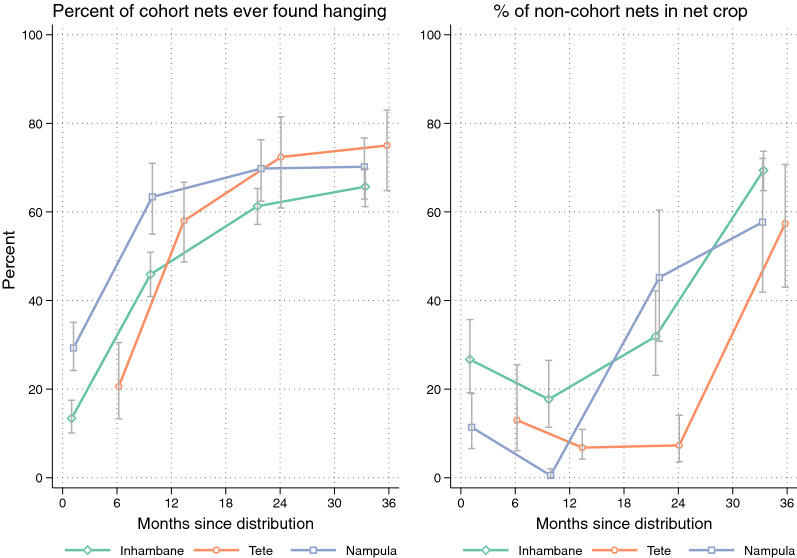
Cohort LLIN found hanging (left) and share of non-cohort LLIN among household net crop (right)

At baseline only 13% of the cohort LLIN in Inhambane were hanging, 21% in Tete and 29% in Nampula and 86%, 68% and 67%, respectively, were still found in the package. Figure 2 (left) illustrates that after 12 months the situation had significantly improved and hanging rates further increased at 24 months finally reaching between 66% in Inhambane and 75% in Tete. At the 36 months survey only between 2% and 4% of the cohort LLIN that were still present in the households were still in the package. Of the cohort LLINs not hanging, some were still being used the previous night, especially in Tete, where 88% of “taken down” LLINs were used and 30% in Nampula. This indicates that they might be removed during the day to gain space in the house. Of nets used the previous night, 81.3% were reported to have been used every night during the previous week. Of the household respondents 81% in Inhambane, 73% in Nampula and 65% in Tete said they used the LLIN equally in the rainy and dry season, but a significant proportion of 18% in Tete also said they used them only during the rains while only 4% in Inhambane and none in Nampula stated this.

In order to interpret the hanging and use of the cohort LLINs, the overall net ownership situation needs to be taken into account and this is shown in Fig. 2 (right). Initially a significant number of non-cohort LLINs were only found in Inhambane and this was due to the fact that during the 2015 campaign some Olyset LLIN had been distributed among the recruited households, but these had not been included in the durability monitoring cohort. At all sites the proportion of households with any non-cohort net and the proportion of these among all LLIN owned by the households declined sharply after baseline suggesting that older, non-campaign LLIN had been discarded. In Nampula new campaign LLIN came in both in 2016 and 2017 (in both cases DuraNet) resulting in an increase to near or above 50% of non-cohort LLIN. In Tete the situation was similar. A campaign that preceded the 36-month survey increased the proportion of non-cohort LLIN within participant households to around 60% (a mix of Olyset and MAGNet). In Inhambane there was a sharp increase of non-cohort LLIN in the 36-month survey (all MAGNet) with non-cohort LLIN reaching a share of 70% of the nets within participant households and these were clearly from the follow up campaign. However, a moderate increase was also seen at the 24-month survey and these LLIN (mostly Dawa Plus) were described in part being from “health facilities” and in part as “from NGO” which could represent the same source.

### Attrition

**Fig. 3:**
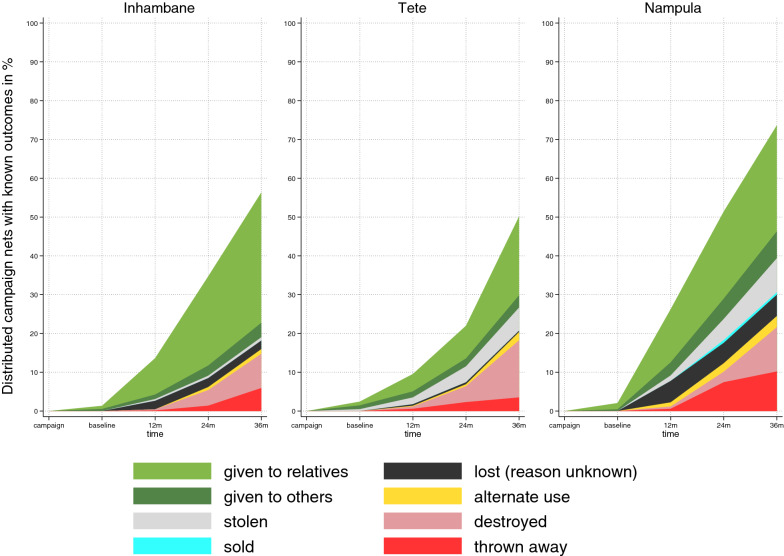
Attrition of cohort LLIN and their causes

The all-cause cohort net attrition rates and losses due to wear and tear (including LLINs that were reported to have been lost between the 2015 campaign and the baseline survey) are shown in Fig. 3. These include only those LLIN for which a definitive outcome could be determined (e.g. if no one was home to be interviewed, or cluster was inaccessible, net status could not be determined). The highest all-cause attrition was seen in Nampula with 74% followed by Inhambane at 56% and Tete with 50%. However, taking into account the different times of observation between Tete and the other two sites as shown in Fig. 3 reveals that all-cause attrition increased more or less linearly at all three sites and was highest in Nampula followed by Inhambane and was lowest in Tete. Attrition due to wear and tear increased in a more curvilinear fashion with very slow increase initially followed by near exponential gains. Attrition due to wear and tear was similar in Inhambane and Tete, but clearly higher in Nampula.

Reasons for loss among the discarded LLIN was similar across the three sites (p = 0.3), with 54% thrown away, 36% destroyed and 10% used for other purposes. Overall there were only 28 cohort LLINs used for other purposes or 2% of all cohort LLINs with a known outcome. Protecting plants was the most commonly reported alternative use in Inhambane and Tete. In Nampula six of the 14 cohort LLINs used for other purposes (1.2% of all LLINs with known outcome) were reported as used for fishing; one was used for drying fish. Other uses were cutting the net up for various uses (two) and as window cover (one).

### Physical condition of observed LLINs

As one would expect, the proportion of LLINs still present in the surveyed households with any sign of damage continued to increase significantly during the monitoring period (Table 4). In Inhambane and Nampula the increases continued up to the final survey while in Tete an equilibrium seems to have been reached where LLINs getting holes and LLINs being discarded occurred at similar rates so that the proportion with any hole no longer increased. The proportion of surviving LLINs that were no longer fit for use due to the level of damage (“torn”) significantly increased at the final survey compared to the modest increases seen previously. In Inhambane it reached 22%, while in Tete it was 36% and in Nampula 37%. This suggests that in Inhambane LLINs were discarded at lower levels of damage compared to Tete as both had similar attrition rates due to wear and tear, but LLIN were much less damaged Inhambane (median pHI among those LLINs with any damage was 269 in Inhambane but 1745 in Tete). In Nampula the proportion of “torn” LLINs was highest and accordingly the proportion of surviving LLINs found in serviceable condition after 3 years was lowest with 63% followed closely by Tete with 64% and Inhambane with 78%.

**Table 4:** Integrity of campaign LLIN present in households

Variable	Baseline	12 months	24 months	36 months
	% (95% CI)	% (95% CI)	% (95% CI)	% (95% CI)
Inhambane	N = 726	N = 589	N = 423	N = 257
Mean months since campaign	1	9.7	21.5	33.4
Net has any hole	2.3 (1.2–4.5)	20.0 (13.6–28.5)	46.8 (40.1–53.6)	58.4 (48.9–67.3)
Physical condition				
Good (0–64)	99.6 (98.2–99.9)	94.2 (91.2–96.2)	77.8 (71.9–82.7)	58.4 (49.7–66.6)
Damaged (65–642)	0.1 (0.0–1.0)	4.2 (2.7–6.7)	15.6 (12.4–19.4)	19.8 (15.2–25.6)
Torn (643+)	0.3 (0.04–2.1)	1.5 (0.7–3.3)	6.6 (3.9–10.9)	21.8 (15.2–30.2)
Serviceable (0–642)	99.7 (97.9–99.9)	98.5 (96.7–99.3)	93.4 (89.1–96.1)	78.2 (69.8–84.8)
Median pHI if any hole (IQR)	23 (2–47)	23 (3–98)	60 (25–290)	269 (51–1193)
Has any repairs if any hole	0 (–.–)	0.8 (0.1–5.4)	12.1 (7.4–19.3)	4.7 (1.9–11.21)
Tete	N = 601	N = 464	N = 306	N = 112
Mean months since campaign	6.2	13.4	24.1	35.8
Net has any hole	7.7 (4.1–13.9)	17.5 (10.3–28.1)	62.8 (50.1–73.8)	58.9 (38.8–76.5)
Physical condition				
Good (0–64)	95.7 (90.8–98.1)	92.0 (82.9–96.5)	58.5 (48.3–68.0)	47.3 (27.2–68.4)
Damaged (65–642)	3.0 (1.4–6.2)	5.2 (2.6–10.0)	22.2 (15.9–30.2)	17.0 (10.3–26.7)
Torn (643+)	1.3 (0.5–3.9)	2.8 (0.9–8.4)	19.3 (11.9–29.7)	35.7 (18.2–58.1)
Serviceable (0–642)	98.7 (96.1–99.6)	97.2 (91.6–99.1)	80.7 (70.3–88.1)	64.3 (41.9–81.8)
Median pHI if any hole (IQR)	137 (23–381)	54 (23–309)	162 (41–1125)	1745 (228–5780)
Has any repairs if any hole	4.3 (1.0–16.8)	7.4 (3.1–16.5)	21.4 (12.3–34.4)	27.3 (12.6–49.3)
Nampula	N = 661	N = 414	N = 268	N = 129
Mean months since campaign	1.2	9.9	21.9	33.3
Net has any hole	0.6 (0.2–1.6)	38.2 (32.3–44.4)	56.3 (43.7–68.2)	88.4 (79.4–93.7)
Physical condition				
Good (0–64)	99.9 (98.8–99.9)	82.6 (78.4–86.5)	68.3 (61.5–74.4)	23.4 (15.4–33.5)
Damaged (65–642)	0.2 (0.0–1.2)	13.8 (10.7–17.5)	23.5 (17.7–30.5)	39.5 (27.7–52.8)
Torn (643+)	0.0 (–.–)	3.4 (2.0–5.7)	8.2 (5.3–12.4)	37.2 (26.7–49.1)
Serviceable (0–642)	100 (–.–)	96.6 (94.3–98.0)	91.8 (87.6–94.7)	62.8 (50.9–73.3)
Median pHI if any hole (IQR)	na	47 (6–226)	98 (29–336)	584 (201–1180)
Has any repairs if any hole	na	10.1 (5.4–18.2)	10.6 (4.6–22.6)	9.6 (4.0–21.5)

### Survival

**Fig. 4:**
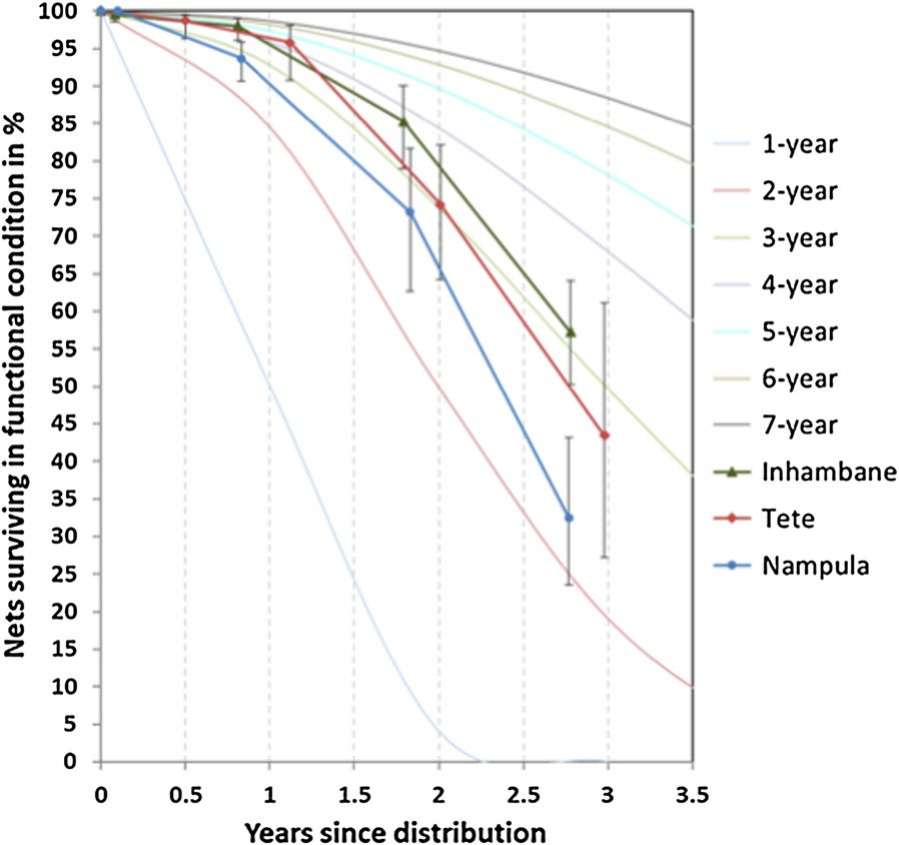
Survival of cohort LLIN in serviceable condition plotted against reference curves with defined median survival

Overall physical survival of LLIN in serviceable condition after 36 months, i.e. the combination of attrition due to wear and tear and the integrity of the still existing LLIN, was 57% in Inhambane, 43% in Tete and 33% in Nampula (Table 5 and Fig. 4). Inhambane performed best and the result was significantly different compared to Nampula (p = 0.0004), but not compared to Tete (p = 0.15). This was due to the higher design effect in Tete of 6.9 (compared to 1.7 in Inhambane and 2.8 in Nampula) which resulted in a very wide confidence interval. In other words, there was a very high variation between communities in durability in Tete.

**Table 5:** Estimated proportion surviving and median survival in serviceable physical condition

Variable	12 months	24 months	36 months
Inhambane			
% surviving in serviceable condition (95% CI)	98.0 (96.0–99.0)	85.3 (78.9–90.0)	57.3 (50.2–64.1)
Estimated from Fig. 4	4.9	3.7	3.1
Calculated from last two data points (95% CI)	–.–	–.–	3.0 (2.8–3.3)
Tete			
% surviving in serviceable condition (95% CI)	95.8 (90.7–98.1)	74.2 (64.2–82.1)	43.4 (27.2–61.1)
Estimated from Fig. 4	4.2	3.1	2.7
Calculated from last two data points (95% CI)	–.–	–.–	2.8 (2.4–3.5)
Nampula			
% surviving in serviceable condition (95% CI)	93.7 (90.6–95.8)	73.2 (62.2–81.9)	32.5 (23.5–43.1)
Estimated from Fig. 4	2.7	2.7	2.2
Calculated from last two data points (95% CI)	–.–	–.–	2.4 (2.1–2.6)

The time of follow-up differed slightly between Tete (35.8 months at the last survey) compared to Inhambane (33.4) and Nampula (33.3). In order to standardize the analysis of survival in serviceable condition, the results were plotted against the hypothetical survival curves with defined median survival (Fig. 4). The survival estimates roughly follow the hypothetical curves and that the relationship between the three sites was the same throughout the time of follow-up.

In addition to estimating median survival at each time point from the graph, it was also calculated from the final two data points (see methods) and results are shown in Table 5. Calculated median survival was 3.0 years in Inhambane (Royal Sentry LLIN), 2.8 years in Tete (MAGNet LLIN), and 2.4 years in Nampula (Royal Sentry LLIN). Estimates obtained from the graph were very similar to the calculated ones at 36 months, but also show that early on in the monitoring the results tend to overestimate the final outcome. Considering the confidence intervals around the median survival, LLINs in Inhambane performed according to the three-year expectation and also in Tete the result was still compatible with the “three-year durability” although given the huge variation between communities in that site this certainly was not true for all villages. In contrast, in Nampula median survival was below the three-year mark.

**Fig. 5:**
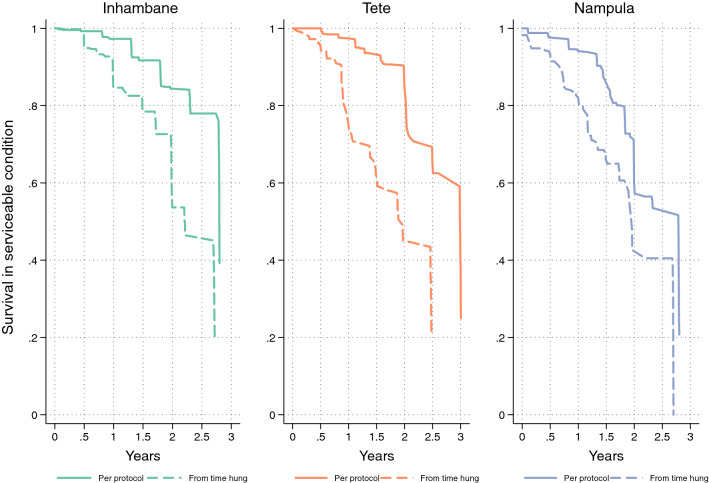
Kaplan-Meier survival functions of cohort LLIN comparing risk starting at distribution versus starting at first hanging

Kaplan–Meier survival curves comparing the intention to treat and per protocol analysis are shown in Fig. 5 and show a similar pattern of survival curves only shifted to the left by 0.2 to 1.0 years when risk of damage is considered to start only when the net is hung for the first time.

Determinants of durability were explored with Cox proportional hazard models. Separate models were constructed for household factors and for net level factors, such that models with net-level factors included only LLIN that had been hung for use during the study. Factors were tested first in individual models then used to construct the final multivariate models. There was interaction between recall of net care messages and net care attitude scores, thus these two variables were combined into a composite variable measuring the intensity of recall and attitudes. Factors that were significantly associated with physical durability were province, message recall/attitudes, household size, cooking in the sleeping room, storing food in the sleeping room, folding up LLIN. Once these were combined in the final multivariate models (Table 6), increased household size was associated with an increased hazard ratio (HR 2.02, p < 0.001 for households with 7 or more people vs those with 1–3 members). Higher net care attitudes and SBC exposure to social and behaviour change (SBC) messages were protective, with moderate attitudes and SBC exposure associated with a nearly 50% reduction in the hazard ratio (p = 0.008) and high attitudes/exposure associated with a nearly two-thirds reduction (p < 0.001). Storage of food in sleeping rooms was not significant in the final model. Compared to Inhambane, Tete was not significantly different, but Nampula had a hazard ratio of 2.25 (p < 0.001), aligning with the overall survival estimates.

**Table 6:** Determinants of physical durability from Cox proportional hazard model

Variable	Adjusted Hazard Ratio (HR)	95% CI	P-value
At household level; N = 5857 obs/2031 LLIN			
Province Inhambane	Reference		
Tete	0.83	0.52–1.31	0.41
Nampula	2.25	1.60–3.17	< 0.001
Household size 1–3	Reference		
4–6	1.30	1.01–1.68	0.045
7+	2.02	1.52–2.69	< 0.001
High net care attitude score and SBC exposure combination across surveys (ref = never (neither at any survey))	Reference		
Moderate (high attitude never or once and SBC exposure at least once)	0.58	0.39–0.86	0.008
Higher (high attitude at least once and SBC exposure at least twice)	0.37	0.24–0.58	< 0.001
Never stored food in sleeping rooms	0.84	0.66–1.06	0.14
At net level (LLIN ever hung) N = 4419 obs/1422 LLIN			
Province Inhambane	Reference		
Tete	0.94	0.58–1.53	0.81
Nampula	2.75	1.88–4.01	< 0.0001
Household Size 1–3	Reference		
4–6	1.22	0.93–1.61	0.15
7+	2.03	1.48–2.79	< 0.0001
High net care attitude score and SBC exposure combination across surveys (ref = never (neither at any survey))	Reference		
Moderate (high attitude never or once and SBC exposure at least once)	0.59	0.40–0.86	0.008
Higher (high attitude at least once and SBC exposure at least twice)	0.39	0.25–0.62	0.0001
Net folded up Always	Reference		
At times	0.56	0.40–0.79	0.001
Never	0.95	0.69–1.31	0.76
Most rudimentary type of sleeping place ever reported for the net (ref = finished bed frame)	Reference		
Unfinished bed frame	0.95	0.62–1.47	0.82
Foam mattress	0.72	0.26–2.01	0.53
Reed mat or ground	1.08	0.79–1.48	0.64

For net-level factors, the final model was similar to the household model but with the addition of folding behaviour and the type of bed frame used with the net. Interestingly, “always” folding up the net was not different from “never” folding up the net, whereas LLINs that had sometimes been folded up had a hazard ratio of 0.56 (p = 0.001). As the vast majority of LLINs in Inhambane and Nampula were never folded up (> 75%) this may be confounded with province. Ultimately the type of sleeping place used with the net was not a significant factor in the final net-level model. Factors that were not significant predictors of physical durability included household head’s age, sex, or education level, discussion of net care in the home, wealth quintile, or the dominant user (adults *vs* adults-children *vs* children only).

### Insecticidal efficacy

The target of sampling 30 campaign LLIN at each site with bio-assay testing was achieved at 12 and 24 months, but at 36 months 30 LLIN were sampled from Inhambane while only 27 each could be obtained from Tete and Nampula. Results of the bioassay testing are shown in Table 7. There was a decline over time of 60-min knock-down percentage at all three sites, with a median of 58% after 36 months in Inhambane and Nampula and 72% in Tete. Decline of vector mortality at the final survey was even more pronounced with a median of 55% in Inhambane, 59% in Tete and 57% in Nampula. As result, optimal insecticidal effectiveness, which was 100% at 12 and 24 months at all sites dropped to just 3% in Inhambane, 11% in Tete and 29% in Nampula at 36 months. However, most samples still achieved the minimal effectiveness threshold with 93% in Inhambane, 100% in Tete and 96% in Nampula meaning that overall only 4% of the 36-month samples must be considered as providing insufficient insecticidal protection. Net handling and use of the sampled LLIN, which were external from the cohort at 12 and 24 months, was comparable to that of the cohort LLIN. This implies that the bio-assay samples can be considered representative of the overall campaign LLIN at these sites.

**Table 7:** Results from bio-assays using WHO cone test

Variable	12 months	24 months	36 months
Inhambane	N = 30	N = 30	N = 30
Knock down 60 minutes			
Mean (95% CI)	97.7 % (96.7-98.7)	88.2 % (86.8-89.7)	68.3 % (66.2-70.5)
Median (IQR)	98.5 % (97.5-99.0)	89.3 % (86.5-90.5)	68.4 % (67.9-69.7)
Mortality 24 hours			
Mean (95% CI)	99.4 % (98.8-99.9)	97.0 % (98.7-99.9)	56.7 % (53.4-60.0)
Median (IQR)	100 % (99.0-100)	98.0 % (95.5-98.5)	55.0 % (52.5-58.5)
Optimal effectiveness			
Estimate (95% CI)	100 % (–, –)	100 % (–, –)	3.3 % (0.4-21.0)
Minimal effectiveness			
Estimate (95% CI)	100 % (–, –)	100 % (–, –)	93.3 % (76.0-98.4)

Variable	12 months	24 months	36 months
Tete	N = 30	N = 25	N = 27
Knock down 60 minutes			
Mean (95% CI)	99.1 % (98.7-99.6)	91.2 % (89.8-92.5)	72.8 % (68.5-77.1)
Median (IQR)	100 % (99.0-100)	89.5 % (87.5-95.5)	71.5 % (65.7-79.5)
Mortality 24 hours			
Mean (95% CI)	98.0 % (96.9-99.1)	97.1 % (95.9-98.3)	63.8 % (59.7-68.0)
Median (IQR)	99.0 % (98.0-100)	98.0 % (96.0-99.0)	58.7 % (56.4-67.8)
Optimal effectiveness			
Estimate (95% CI)	100 % (–, –)	100 % (–, –)	11.1 % (3.8-28.4)
Minimal effectiveness			
Estimate (95% CI)	100 % (–, –)	100 % (–, –)	100 % (–, –)

Variable	12 months	24 months	36 months
Nampula	N = 30	N = 30	N = 27
Knock down 60 minutes			
Mean (95% CI)	98.2 % (97.1-99.3)	90.8 % (89.8-91.9)	66.5 % (59.8-73.2)
Median (IQR)	98.0 % (98.0-100)	90.5 % (89.0-93.5)	57.9 % (52.1-89.5)
Mortality 24 hours			
Mean (95% CI)	98.8 % (98.4-99.1)	97.0 % (96.3-97.7)	68.3 % (60.9-75.8)
Median (IQR)	99.0 % (98.0-100)	97.0 % (96.5-98.0)	56.7 % (53.3-94.5)
Optimal effectiveness			
Estimate (95% CI)	100 % (–, –)	100 % (–, –)	29.3 % (15.4-49.4)
Minimal effectiveness			
Estimate (95% CI)	100 % (–, –)	100 % (–, –)	96.3 % (77.3-99.5)

Chemical testing of the 36-month samples found that median g/kg was 4.70 for Inhambane (81% of target dose), 2.36 for Tete (42% of target dose), and 1.85 for Nampula (32% of target dose).

## Discussion

Overall results indicate a median physical survival of 2.2 years in Nampula (Royal Sentry^®^), 2.7 years in Tete (MAGNet^®^), and 3.1 years in Inhambane (Royal Sentry^®^) for the 150-denier polyethylene LLIN. Differences in survival appear to be driven primarily by site-level differences in household and net environment.

Reasons for loss of LLINs were similar across the three sites. Cohort LLINs were given away primarily to relatives in all sites and this comprised the greatest share of all-cause attrition. Loss due to wear and tear was limited until 24 months when it accelerated sharply in all three sites, but to a greater extent in Nampula. In addition to the increasing age of the LLIN, this is likely also related to the influx of new campaign LLINs particularly in Nampula but also in the other sites prior to the 36-month round. LLINs sold, stolen, or used for other purposes were a relatively small share of all-cause attrition, similar to continent-wide findings [16]. LLIN were discarded earlier in Inhambane, while in Tete and Nampula households tended to hold on to them longer.

Physical survival and attrition findings are in line with an earlier durability study conducted in Nampula from 2008 to 2011 [10], although a few key methodological differences should be noted. In the 2008 study, LLINs were tagged prior to distribution, and then tagged LLINs were identified during a post-campaign hang up exercise, and a single net per household followed up and removed for additional testing. Similar to the present findings, the most common cause of attrition in the first 2 years was giving away, or stolen, and only 5% of LLIN in the first year and 34.7% in the second year were lost due to wear and tear. Rodent damage accounted for 20.5% of the times a net was discarded and was the most commonly reported cause of damage generally, in line with the present findings about rodents in Nampula.

Inhambane had a markedly higher socioeconomic status than the other two regions. Hanging and use of the LLIN was similar across the three sites. The practice of folding LLIN up during the day, which has been shown to be protective against damage in other settings [17, 18], was poor overall but more common in Tete, where most sleeping places were reed mats. Cooking in sleeping rooms was relatively rare in all sites, and storage of food in sleeping rooms (associated with rodent presence and rodent damage to LLIN) was rare in Inhambane and Tete but much more prevalent in Nampula. Washing and drying behaviour were similar across sites, with wash frequency roughly once every 3 months, and drying on bushes or fencing more common in Tete.

In Nampula, the shorter median lifespan noted in the present study may have been influenced by the influx of new campaign LLIN or by the net handling risk factors there. Although there was no direct evidence for this from the rate of increase of all-cause attrition, it cannot be excluded that median lifespan might have been longer had there not been additional rounds of campaigns. Given the relatively poor recall of net care messages, low levels of positive net care attitudes, and moderate rates of preventive net handling behaviours such as tying LLIN up during the day, there is certainly room to improve some specific behaviours through social and behaviour change messaging, which has been shown to be associated with a 6-month longer median lifespan in Nigeria [19]. Changing the household environment—incl. storing food in sleeping rooms, bed frames—is more difficult.

Results from the Cox model suggest that unmeasured province-level differences were the primary factor driving the shorter median lifespan in Nampula. Hazard ratio also increased with increasing household size. Larger households have more children, and children (particularly those under 5 years old) have been previously shown to be associated with damage to LLINs [17]. These findings may also reflect the difficulty of maintaining good care of LLINs in households with multiple children. Larger households are less likely to have enough LLINs for every member [20], and therefore children may be sharing LLIN to a greater degree, potentially putting more strain on the LLIN during nightly use, in addition to daytime play that might cause damage. Positive attitudes towards care and repair combined with recall of net care SBC were increasingly protective, suggesting that expansion of net care messaging may help more LLIN in Mozambique last longer, potentially maintaining higher rates of LLIN access. The attitudes measured in this monitoring reflect self-efficacy to care for and repair LLINs, perceived effectiveness of LLIN, and the social norm of net repair. Surprisingly, the practice of folding LLINs up during the day, shown to be very protective in other contexts [17, 18], was protective only for LLINs that were ‘sometimes’ folded up, compared to LLIN that were always or never folded; however, as nearly 70% of nets were never folded and very few (16%) were ‘always’ folded this may reflect a sample size issue. The type of sleeping place used with the net was not significant in the final model, suggesting that protective behaviours can overcome challenging household environments.

Insecticidal efficacy against susceptible mosquitoes was optimal through 12 and 24 months but dropped precipitously at 36 months. However, the majority of LLINs were still minimally effective at 36 months, even as chemical content ranged from 32 to 81% of target dose. This compares favourably to Madagascar, where mosquito mortality for Royal Sentry fell from 90% at baseline to 23% at 12 months, but with significant variation between sites [21]. Phase 2 trials in India indicated that MAGNet retained 100% mortality in cone bioassays after 25 washes [22], and in Burkina Faso washing MAGNet 20 times resulted in a 40% reduction in protection against pyrethroid-resistant *Anopheles gambiae* sensu lato blood feeding [23]. With relatively little data published for MAGNet and Royal Sentry, additional monitoring of these nets would improve the evidence base.

### Limitations

As with all household surveys, there is a potential for response bias, although questionnaires were designed to minimize this. With the prospective design, there is also the potential for the Hawthorne effect, whereby being asked about net care and handling four times over the course of 3 years may have contributed to changes in behaviour. The standard durability monitoring approach tries to minimize this by conducting only baseline and annual surveys vs every 6 months as had been done in some of the earlier studies. The inability to access some clusters in certain rounds and in particular the fabrication of data in two clusters in Tete at 36 months in combination with a higher than expected loss to follow-up resulted in high confidence intervals for the survival estimates in Tete and reduced the study’s power to detect differences with the other sites. However, this did not affect comparisons between the Nampula and Inhambane sites.

## Conclusion

After 3 years of follow-up among rural populations in the provinces of Inhambane, Tete and Nampula, the 150-denier polyethylene LLIN Royal Sentry^®^ and MAGNet^®^ showed significant differences in median physical survival ranging from 3.1 years in Inhambane to 2.7 in Tete and 2.2 in Nampula. These differences could be attributed at least in part to household environment and net care and repair behaviours. This means that in two of the three sites the assumption of a three-year cycle of campaign distributions holds, while in the Nampula site either additional continuous distribution channels could be added and/or existing channels bolstered, and/or more intense or targeted social and behaviour change activities to encourage net care and retention could be considered. Insecticidal performance was optimal as tested by bio-assay for 100% of samples up to 24 months follow-up, but declined at 36 months. In Inhambane, only 3% of samples showed optimal effectiveness at 36 months, compared with 11% in Tete and 29% in Nampula. However, most LLIN (96% overall) still had minimal effectiveness and hence provided at least some level of protection. As LLIN with synergist or dual active ingredients become more common, assays to monitor their effectiveness against resistant mosquitoes are urgently needed and should be integrated into standard durability monitoring approaches.

## Supplementary information

**Additional file 1.** Household characteristics (based on households that were seen at baseline and endline surveys).

## Data Availability

The datasets used and/or analysed during the current study are available from the corresponding author on reasonable request.

## Abbreviations

CDC: Centers for Disease Control and Prevention
CIPAC: Collaborative International Pesticides Analytical Council
GPS: Global Positioning System
JHU: Johns Hopkins University
LLIN: Long-lasting insecticidal net
NMCP: National Malaria Control Program
NIH: National Institute of Health
ODK: Open Data Kit
OSMAND: Open Street Map for Android
pHI: Proportionate hole index
PMI: U.S. President’s Malaria Initiative
SBC: Social and behaviour change
USAID: United States Agency for International Development
WHO: World Health Organization
WHOPES: World Health Organization Pesticide Evaluation Scheme
