# Supplementary material for: Monitoring the durability of the long-lasting insecticidal nets MAGNet and Royal Sentry in three ecological zones of Mozambique
Source: Malar J. 2020 Jun 17;19:209. doi: 10.1186/s12936-020-03282-w (PMC7301518; doi:10.1186/s12936-020-03282-w)
Supplement: Supplementary file 1 — Additional file 1. Household characteristics (based on households that were seen at baseline and endline surveys). [file 12936_2020_3282_MOESM1_ESM.docx]

# Additional Files

## Additional file 1

Household characteristics (based on households that were seen at baseline and endline surveys)

| **Variable** | **Inhambane** | **Tete** | **Nampula** | **P-value**  for comparison between sites |
| --- | --- | --- | --- | --- |
|  | % (95% CI) | % (95% CI) | % (95% CI) |  |
| **Demographic** |  |  |  |  |
| Mean number of de-jure members | 5.0 (4.7-5.4) | 4.0 (3.4-4.7) | 4.1 (3.5-4.7) | 0.004 |
| Mean age of head of household in years | 48.8 (46.7-51.0) | 44.0 (40.7-47.4) | 44.4 (42.0-46.8) | 0.007 |
| Proportion of female headed households | 22.5 (16.7-29.6) | 15.9 (10.5-23.3) | 12.8 (8.4-19.1) | 0.05 |
| Proportion of under-fives | 12.1 (10.8-13.5) | 14.1 (12.1-16.4) | 11.4 (9.0-14.2) | 0.22 |
| Education of male heads of household  Non-literate  Primary  Secondary | 25.5 (19.6-32.5)  49.8 (41.9-57.8)  21.6 (15.7-29.1) | 50.4 (42.7-58.0)  37.5 (32.1-43.2)  11.0 (5.6-20.4) | 52.0 (43.0-60.9)  37.3 (30.7-44.3)  10.7 (5.6-19.3) | 0.001 |
| **House characteristics** |  |  |  |  |
| Improved roof materials | 4.9 (3.0-8.0) | 0 | 0 | 0.002 |
| Improved wall materials | 42.5 (36.5-48.9) | 24.2 (12.3-42.1) | 20.5 (11.3-34.3) | 0.02 |
| Improved floor materials | 77.8 (68.2-85.1) | 14.4 (4.8-35.7) | 18.2 (9.2-33.0) | 0.000 |
| Cooking fuel firewood or charcoal | 98.6 (96.3-99.5) | 99.6 (97.3-100.0) | 100.0 | 0.09 |
| **Water and sanitation** |  |  |  |  |
| Access to safe water | 100.0 | 78.8 (47.1-93.9) | 89.0 (76.1-95.4) | 0.04 |
| Access to any latrine | 97.3 (94.0-98.8) | 75.4 (62.2-85.1) | 67.9 (53.0-79.9) | 0.000 |
| Improved latrine or flush toilet | 45.5 (36.1-55.3) | 14.4 (6.5-28.8) | 4.0 (1.9-8.6) | 0.000 |
| **Household assets** |  |  |  |  |
| Any transport | 37.6 (30.4-45.3) | 49.6 (38.0-61.2) | 65.0 (55.6-73.4) | 0.0002 |
| Type of transport  Bicycle  Motorcycle  Car | 28.0 (21.3-35.8)  5.7 (3.1-10.3)  10.0 (6.6-14.8) | 40.5 (30.5-51.4)  9.8 (6.1-15.5)  1.1 (0.3-4.8) | 52.3 (40.1-64.3)  18.2 (12.2-26.2)  0.6 (0.1-4.1) | 0.002  0.001  0.000 |
| Owns farm land | 95.5 (90.9-97.8) | 83.7 (65.4-93.3) | 82.1 (67.7-90.9) | 0.04 |
| Owns livestock | 91.0 (87.3-93.7) | 67.8 (60.6-74.3) | 55.5 (47.1-63.6) | 0.000 |
| Type of livestock  Chicken  Goats  Cows | 80.6 (74.4-88.5)  32.0 (23.0-42.5)  15.1 (11.0-20.3) | 47.4 (41.5-53.5)  44.2 (34.6-54.3)  39.5 (30.3-49.4) | 43.6 (34.3-53.4)  11.5 (7.1-18.1)  7.4 (3.9-13.4) | 0.000  0.000  0.000 |
| Household items owned  Radio  Television  Refrigerator  Fan  Iron  Any mobile phone  Smartphone or computer | 62.0 (55.1-68.5)  40.6 (32.1-49.7)  17.4 (10.2-27.9)  11.8 (7.2-18.9)  12.5 (6.5-22.4)  71.2 (61.3-79.4)  18.2 (12.6-25.4) | 40.5 (30.1-51.9)  7.2 (1.4-29.1)  5.3 (0.7-31.0)  2.7 (0.4-17.1)  4.9 (0.9-22.8)  28.8 (20.1-39.5)  3.8 (1.6-8.8) | 43.1 (34.5-52.1)  14.5 (7.0-27.6)  5.2 (1.8-14.0)  3.5 (1.3-8.7)  5.5 (1.9-15.0)  57.5 (47.4-67.0)  1.2 (0.4-3.6) | 0.0008  0.0014  0.12  0.05  0.29  0.000  0.000 |
